# Detection of Influenza D Virus among Swine and Cattle, Italy

**DOI:** 10.3201/eid2202.151439

**Published:** 2016-02

**Authors:** Chiara Chiapponi, Silvia Faccini, Aurora De Mattia, Laura Baioni, Ilaria Barbieri, Carlo Rosignoli, Arrigo Nigrelli, Emanuela Foni

**Affiliations:** Istituto Zooprofilattico Sperimentale della Lombardia ed Emilia Romagna, Brescia, Italy (C. Chiapponi, S. Faccini, A. De Mattia, L. Baioni, I. Barbieri, C. Rosignoli, A. Nigrelli, E. Foni);; World Organisation for Animal Health Reference Laboratory for Swine Influenza, Parma, Italy (C. Chiapponi, L. Baioni, E. Foni)

**Keywords:** Influenza D virus, IDV, genome, zoonoses, cattle, swine, cows, pigs, Italy, D/swine/Oklahoma/1334/2011, viruses, influenza, respiratory infections

**To the Editor**: Recent studies have identified a new genus of the *Orthomyxoviridae* family (*1*–*5*). The virus, distantly related to human influenza C virus, has been provisionally designated as influenza D virus. This novel virus was identified for the first time in pigs with influenza-like illness (*1*), but subsequent serologic and virologic surveys have suggested cattle as a possible reservoir (*2*–*4*). Moreover, the virus was shown to infect ferrets used in laboratories as surrogates for humans when investigating influenza infection (*1*). In a serologic study conducted on 316 human samples, low antibody titers and a low level of positive samples (1.3%) were detected (*1*), suggesting that humans are a possible host to be studied in depth. To investigate the circulation of influenza D viruses among pigs and cattle in Italy, we performed biomolecular and virological tests on clinical samples collected from respiratory outbreaks in Po Valley, the area in Italy with the highest density of swine and cattle farms.

We screened clinical specimens from swine (n = 150) and cattle (n = 150) for influenza D virus by reverse transcription quantitative PCR (*1*). Three nasal swab samples were found positive: 1 from a sow and 2 from cattle, collected from 3 farms located at linear distances ranging from 47 to 80 km. All positive samples were confirmed by partial polymerase basic 1 gene sequencing and submitted to viral isolation in cell cultures as previously described (*5*,*6*). The virus was isolated on CACO-2 and HRT18 cell cultures only from the sow sample (D/swine/Italy/199723-3/2015). Cell cultures were tested by using reverse transcription quantitative PCR. Viral RNA was isolated from clinical samples or cell culture by using One-For-All Vet Kit (QIAGEN, Milan, Italy). Full-genome amplification from influenza D virus–positive samples was achieved as previously described (*3*). A sequencing library of the purified amplicons was prepared by using NEXTERA-XT kit and sequenced by using a Miseq Reagent Kit v2 in a 250-cycle paired-end run (both from Illumina Inc., San Diego, CA, USA). Sequencing reads were assembled de novo or by using D/swine/Oklahoma/1334/2011 (GenBank accession nos. JQ922305–JQ922311) as a template by Seqman NGen DNASTAR version 11.2.1 (DNASTAR, Madison, WI, USA). Gene sequences from the 3 influenza D viruses isolated in Italy and all the available influenza D virus sequences retrieved from GenBank were aligned with ClustalW by using MEGA5 (*7*). We analyzed the predicted amino acid sequences for each gene. 

Phylogenetic trees of the individual segments were inferred by using the maximum-likelihood method implemented in the IQ-TREE package 0.9.6 (*8*). The robustness of the maximum-likelihood trees was evaluated by bootstrap analysis by comparison to 1,000 bootstrap samples. The swine isolate D/swine/Italy/199723-3/2015 was fully sequenced (GenBank accession nos. KT592530–KT592536). Full genome sequences of D/bovine/Italy/1/2014 (GenBank accession nos. KT592516–KT592522) and D/bovine/Italy/46484/2015 (GenBank accession nos. KT592523–KT592529) were obtained directly from the nasal swab samples. The 7 genomic segments of each of the 3 influenza D virus genomes encode the proteins of polymerase basic subunits 1 and 2, polymerase 3, glycoprotein, nucleoprotein, matrix 1, matrix 2, and nonstructural proteins 1 and 2. These segments contain 772, 755, 710, 664, 552, 387, 246, 243, and 184 aa residues, respectively, similar to viruses of their counterparts of the isolates documented in Asia and America. The predicted amino acid sequence of the hemagglutinin gene shows unique features for the strains isolated in Italy: V in position 289, K409R, I563L, and A652V. In the apex of the hemagglutinin 1 receptor-binding domain of the glycoprotein-predicted proteins, position 212 is occupied by K, as previously observed for D/swine/Oklahoma/1334/2011 (*5*). Moreover, the 3 isolates from Italy share unique mutations in the polymerase basic 1 gene (R191G, F278S, R444G) and in the polymerase 3 predicted proteins (I194V, M596V). D/swine/Italy/199723-3/2015 shows no unique amino acid difference to bovine strains, and its gene segments cluster with influenza D viruses isolated from cattle, suggesting the circulation of this virus among cattle and swine in Italy. Phylogenetically, all 7 segments of the strains isolated in Italy clustered with D/swine/Oklahoma/1334/2011, showing no sign of reassortment (Figure).

**Figure:**
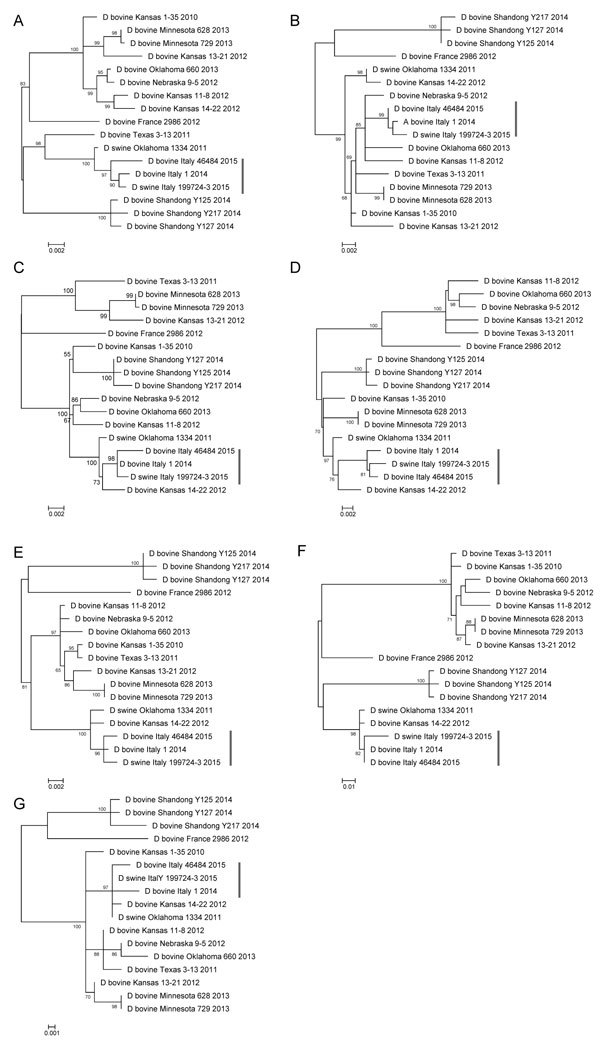
Phylogenetic trees of the 7 genes of influenza D viruses obtained from 1 sow and 2 cattle in Italy (vertical bars) and comparison isolates retrieved from GenBank. A) Polymerase basic (PB) 2: 2,319 nt; B) PB1: 1,434 nt; C) P3: 2,133 nt; D) glycoprotein hemagglutininesterase: 1,995 nt; E) nucleoprotein: 1,659 nt; F) polymerase 42: 1,164 nt; G) nonstructural: 732 nt. Genes were trimmed and aligned, then phylogenetically analyzed by using the maximum-likelihood method. Sequences are listed by their host, country, strain name, and collection year. Scale bars indicate nucleotide substitutions per site.

Our findings show that influenza D viruses circulate among swine and bovine herds in Italy affected by respiratory disease. Genetic analysis highlights that the swine and bovine influenza D viruses are very closely related, both belonging to the D/swine/Oklahoma/1334/2011 cluster. Further studies are ongoing to better understand the epidemiology, virology, and pathobiology of influenza D virus in swine and cattle, especially concerning the evidence that Koch’s postulates are fulfilled for this agent. Implications in zoonotic aspects of influenza D virus infections will be also considered in ongoing research.
